# Reply to: Surgical scene understanding and the emerging challenge of independent validation in an industry-led AI ecosystem

**DOI:** 10.1038/s41746-026-03028-z

**Published:** 2026-08-10

**Authors:** Matthias Carstens, Shubha Vasisht, Zheyuan Zhang, Iulia Barbur, Annika Reinke, Lena Maier-Hein, Daniel A. Hashimoto, Fiona R. Kolbinger

**Affiliations:** 1 Weldon School of Biomedical Engineering, Purdue University, West Lafayette, IN USA; 2 Department of Visceral, Thoracic and Vascular Surgery, University Hospital and Faculty of Medicine, TUD Dresden University of Technology, Dresden, Germany; 3 Department of Surgery, University of Pennsylvania Perelman School of Medicine, Philadelphia, PA USA; 4 Division of Intelligent Medical Systems (IMSY), German Cancer Research Center (DKFZ), Heidelberg, Germany; 5 Helmholtz Imaging, German Cancer Research Center (DKFZ), Heidelberg, Germany; 6 Surgical AI Research Group, Heidelberg University Hospital, Heidelberg, Germany; 7 National Center for Tumor Diseases (NCT) Heidelberg, Heidelberg, Germany; 8 Faculty of Mathematics and Computer Sciences, Heidelberg University, Heidelberg, Germany; 9 Mohamed Bin Zayed University of Artificial Intelligence (MBZUAI), Abu Dhabi, United Arab Emirates; 10 Department of Computer and Information Science, University of Pennsylvania School of Engineering and Applied Science, Philadelphia, PA USA; 11 Else Kröner Fresenius Center for Digital Health, Medical Faculty and University Hospital Dresden, TUD Dresden University of Technology, Dresden, Germany

**Keywords:** Business and industry, Computational biology and bioinformatics, Mathematics and computing, Medical research, Scientific community, Social sciences

## Abstract

In this reply to MacAonghusa and Cahill, we reflect on fundamental validation challenges in AI-enabled surgical scene understanding and propose key priorities that academic, clinical, and industry stakeholders must collaboratively address to achieve safe and impactful clinical translation of surgical video AI: First, tying descriptive analytics to outcomes that matter to patients or surgical teams, second, establishing longitudinal, multimodal, and outcome-linked data infrastructure, and third, implementation research.

We thank MacAonghusa and Cahill for their thoughtful Matters Arising, which extends the scope and discussion of our systematic review in a clinically important direction. Their central observation that digital surgery innovation is increasingly driven and made available to clinical teams via commercially available platforms that are incompletely represented in the peer-reviewed literature is well-supported. We appreciate the opportunity to discuss the implications of these parallel trajectories of academic and industry-led teams for validation and clinical translation of surgical video AI.

Our systematic review and reporting quality meta-analysis^1^ was scoped to the published academic literature. Academic literature reviews are inherently limited to published evidence. The commercial surgical scene understanding (SSU) landscape follows a parallel, and in important respects divergent, trajectory that is often driven by clinical and administrative needs rather than by discovery and independent evidence generation. In contrast to most academic publications included in our systematic review, many commercial platforms do not focus exclusively on SSU capabilities, such as anatomy recognition. Instead, they combine AI-enabled surgical video analytics (of which SSU is one component) with administrative and operational functions such as video logging, clinical database management, and workflow documentation (Table 1). This bundled structure complicates benchmarking efforts and comparison with experimental SSU capabilities presented in academic works. At the same time, the adoption of commercial platforms in operating room settings does not equate to evidence of SSU-specific clinical benefit. These contemporary deployment realities conflict with traditional academic standards for validation of medical innovation.

**Table 1:** Examples of commercially available and emerging digital surgical platforms with surgical scene understanding capabilities

Manufacturer	Platform	Key platform functions	Key publicly available evidence on platform performance
Anaut Inc.	EUREKA X	• Anatomical mapping • Bleeding tracking • Instrument tracking	• AI-enabled nerve recognition in 51 laparoscopic colorectal surgeries: overall nerve recognition failure rates: 26–64%, AI assistance improved identification up to 91%^17^ • Segmentation of loose connective tissue in 33 gastric cancer surgeries: mean recall: 0.61, mean F1-score: 0.55, high sensitivity rating by expert surgeons^18^ • Anatomical mapping in 150 TAPP surgery videos: mean IoUs for connective tissue: 0.33, nerves: 0.24, vas deferens: 0.50, microvessels: 0.30^19^
Intuitive	MyIntuitive	• Surgical video capture and storage, structured video library • Surgical phase identification • Personalized summary of procedural phase duration, instrument usage, performance comparisons against historical trends or anonymized benchmarks • Integrated training modules and statistics	• Proficiency gains after implementing telemetry functionality: Decreases in idle time (42 to 32 min), console time (470 to 395 min), conversion rate (23.3 to 7.5%) after about 60 robotic pancreatic surgeries^20^ • Association of MyIntuitive app use with increased case volumes^21^
Medtronic	Touch Surgery Enterprise	• Automated surgical video capture and storage, structured video library • Recognition of procedure phases/steps across surgical specialties • Recognition of anatomy and instrument time in view • Performance and quality analytics (timing, camera out time, phase timing metrics, and comparison with global trends) • Surgical workflow integration	• Manually graded case difficulty related to platform-measured operative time and manually graded CVS achievement in 206 laparoscopic cholecystectomy videos: grade 1–2 cases (69%) had a median 17:53 min vs grade 3–4 (31%) 25:49 min operative time; CVS was achieved in 91% grade 1, 86% grade 2, 83% grade 3, and 73% grade 4 cases^22^ • Improved skill ratings across 59 laparoscopic suturing task videos for platform users scoring 89.7% on tendon repair simulation, compared to textbook users who scored 63.4%^23^
Oath Surgical	OathOS	• Clinical documentation, scheduling, and preoperative workflow planning • Real-time analysis of surgical video and audio for workflow optimization (e.g., procedure time, identifying steps, instruments, and sponge count) • Database tools connecting postoperative outcomes, recovery metrics, and patient-reported measures with the procedure • Reporting of surgeon performance metrics (e.g., average surgery duration, facility savings, complication rate)	• No peer-reviewed or manufacturer-published performance data identified
Proximie	Proximie Platform	• Surgical video capture and storage, structured video library • Real-time remote access, live streaming, and collaboration with other users • Real‑time AI‑enabled dashboards and notifications to optimize workflows by identifying surgical events and key workflow milestones from video and device data	• No peer-reviewed or manufacturer-published AI performance data identified • Educational experience of live-streaming functionality evaluated by medical students^24^
Scialytics	Scialytics Assist	• Surgical video capture and storage, structured video library • Real-time intraoperative AI guidance for laparoscopic cholecystectomy: detection of critical surgical steps and landmarks (e.g., cystic duct, cystic artery), alerts for potential safety risks during dissection • Automated annotation of surgical video for documentation and review	• No peer-reviewed or manufacturer-published performance data identified
SurgeonAI	SurgeonAI Platform	• Real-time safety alerts, anatomical guidance, and quantitative information aimed at preventing errors during minimally invasive surgery	• No peer-reviewed or manufacturer-published performance data identified
Surgical Data Science Collective	Surgical Video Platform	• Secure, cloud-based library for surgical videos • Tools for timestamped commentary • Surgical phase breakdown • Instrument recognition and temporospatial analytics of instrument use	• Surgical phase identification of endoscopic pituitary tumor surgeries, tested on 127 video clips across 38 case videos (accuracy: 77.7%)^25^
Theator	Theater Surgical Intelligence Platform	• Automated surgical video capture and storage, structured video library • AI-generated operative reports (prepopulated text matched to scene characteristics), billing documentation • Surgeon, department, and system-level dashboards from intraoperative video with variability tracking, benchmarking, and outcome correlation • Procedure time analysis, complexity scoring, phase/step duration tracking • Searchable, AI-annotated case library, technique comparisons, resident assessments, indexed by step, event, complexity	• Operative reports: Higher accuracy for AI-supported operative reports compared with surgeon operative reports (87.3 vs 72.8%) in 158 cases^26^ • Increased CVS achievement (39.2 to 69.2%) in 279 laparoscopic cholecystectomies after platform introduction^27^ • Performance analytics: Capture rate 96.7% across 11,080 minimally invasive surgeries, detection of key steps (e.g., CVS achieved in 60.6% of 2136 cholecystectomies), analysis of inter-center variability for CVS achievement (14–70%), surgeon self-review in 22.2% of cases^28^ • Surgical step identification in 277 laparoscopic hysterectomies (accuracy: 93.1%)^29^ • Identification of warm ischemia times in 61 cases of partial nephrectomies: accuracy: 80–97%^30^ • Real-time annotation of safety-related SSU timepoints in 2 robot-assisted urologic surgeries (qualitative visual representation of predictions)^31^

Platforms were identified through a targeted web search via Google and from the *Artificial Intelligence (AI)-Enhanced Surgical Video Analytics Global Market Report 2025*^32^. Key platform functions and corresponding performance evidence were gathered from publicly available sources, including product information from manufacturers. Given the heterogeneity of these sources (including non-peer-reviewed references), the table constitutes a scoping overview of claimed platform functions rather than an evidence-based assessment of validated clinical performance. Sources that do not explicitly link evidence to a commercially available or emerging digital surgical platform were not included.

*CVS* critical view of safety, *SAGES* Society of American Gastrointestinal and Endoscopic Surgeons, *TAPP* Transabdominal Preperitoneal Hernia Repair.

Benchmarking and validation efforts are generally demanded by regulatory laws governing medical device deployment. These regulations are intended to ensure patient safety and objectively evaluate the clinical benefits that an innovation offers to patients and surgical teams. This raises the question of how meaningful validation for surgical video AI tools can be designed. A fundamental challenge for the field is the fuzzy relationship between the (largely descriptive) tasks that current surgical video AI tools address, and objective, clinically relevant endpoints that matter for patients or surgical teams. Surgical endpoints that reflect patient safety, operative quality, or healthcare system efficiency may include complication rates, operative time, conversion rates, or cost, as opposed to technical surrogate metrics, such as detection accuracy or frame-level segmentation performance. However, unlike many diagnostic AI applications, where a prediction task, e.g., detecting a suspicious lesion on cancer screening mammograms, maps relatively directly onto a clinical decision and a measurable outcome, SSU tasks are more challenging to evaluate against clinical outcomes. For example, an AI model that detects the critical view of safety (CVS) during laparoscopic cholecystectomy does not straightforwardly prevent bile duct injury. Bile duct injury may occur even when the CVS is achieved, and it is avoided in many cases where it is not. This disconnect between observable intraoperative scene characteristics and behaviors, and objectifiable clinical outcomes means that purely descriptive tasks, for which technical metrics such as detection accuracy might suffice, cannot serve as adequate metrics for clinical utility. Yet, this is precisely what the field has defaulted to, both in academic research, as our review documents, and in commercially deployed systems, which often remain poorly contextualized with respect to clinical utility and lack rigorous independent validation against clinically meaningful endpoints (Table 1). The consequence is that neither existing academic benchmarks nor the descriptive outputs generated by many industry platforms adequately assess whether SSU tools improve outcomes that matter to patients or surgeons, or result in administrative healthcare system-level improvements. At the same time, published evidence strongly supports the use of spatiotemporal visual cues from surgical video to model clinical outcomes^2–4^, yet the automation of identifying such cues has long been a bottleneck. A recent study reports end-to-end prediction of postoperative erectile function recovery after robot-assisted radical prostatectomy via gesture patterns from pre-selected surgical video sections using a spatiotemporal transformer model^5^. This study represents a relevant step towards meaningful outcome prediction from surgical video, as the approach may be transferable to other procedures and is suitable for independent, prospective validation.

To overcome the outlined conceptual barriers related to validation and accelerate progress in the field, we would like to highlight three fundamental priorities that academic, clinical, and industry stakeholders will need to collaboratively address. First, *surgical procedures need to be foundationally characterized in terms of how parameters recognizable or measurable from surgical video relate to objective, relevant clinical outcomes*, to inform outcome-linked validation strategies for surgical video AI models^6^. Notably, this association between video-derived cues and clinical outcomes is not direct, as it is impacted by model-inherent inaccuracies in identifying these cues, and confounding patient- and disease-related factors. Examples of likely associations include links between tissue-handling behaviors and histopathologic specimen quality in colorectal cancer surgery^7^, or between pancreatic capsule disruption or staple line bleeding during pancreatic transection and postoperative complications after pancreatic surgery^8,9^.

To achieve this characterization, a second priority is the *implementation of appropriate data infrastructure* as a clinical standard. This infrastructure needs to efficiently and reliably link intraoperative video data with relevant multimodal data (e.g., preoperative routine imaging, laboratory parameters), clinical metadata (e.g., age, sex, comorbidities), and outcomes, along the treatment pathway. If equipped with appropriate data management tools, academic and clinical stakeholders are well-positioned to drive this infrastructure development, given their access to patient data, outcome records, and the clinical expertise needed to define what outcomes matter. Realizing this infrastructure will require deliberate strategies to ensure patient privacy and adequate data governance without compromising research integrity and may, for example, leverage decentralized learning^10^ and synthetically generated surgical video data to augment limited real-world datasets^11^. To ensure data privacy for video recordings for both patients and surgeons, the scientific community and many industry stakeholders have adopted standards for video data deidentification^12^. At the same time, many practitioners and clinical institutions desire long-term protection against medicolegal scrutiny based on outcome-linked video findings, yet existing strategies that are part of commercial platforms offer only vague protection, if any. Ultimately, linking video data and outcomes may be feasible at scale only with specific governance models that both protect industry interests and enable independent oversight. One possible approach could be the use of trusted third-party structures, such as certified academic consortia or public health institutions acting as neutral data custodians, which could support multimodal data linkage. Controlled access frameworks, where access is limited to verified researchers working within clearly defined analytical boundaries, could facilitate academic research and discovery on these valuable datasets, similar to large-scale population health databases like the UK Biobank^13^ and the All of Us research program^14^.

Third, *implementation research* needs to become a scientific priority. This encompasses human-AI interaction research (understanding how clinicians interpret and act on SSU outputs in real time) as well as the development of regulatory structures that enable controlled, prospective evaluation of deployed systems. Here, we share the concern raised by MacAonghusa and Cahill about the structural asymmetry between the regulatory burden placed on academic researchers seeking to rigorously evaluate Software as a Medical Device tools and the comparatively permissive conditions under which commercial platforms are deployed, updated, and monitored after deployment. These asymmetries operate on two levels: while industry stakeholders can iteratively deploy and update platforms, often retaining privileged access to large-scale real-world data that enables continuous post-deployment evaluation and performance monitoring, academic and investigator-initiated teams face disproportionate upstream barriers to conducting independent validation studies. These include restricted access to patient data and fragmented regulatory requirements. As a concrete example, in the European Union, heterogeneous national interpretations of Article 82 of the Medical Device Regulation mean that low-burden observational studies may face requirements equivalent to full clinical investigations in some Member States, which places a disproportionate burden on academic teams without dedicated regulatory infrastructure^15^. On a second level, there is also a need for regulatory frameworks to make post-deployment monitoring feasible for independent academic teams. Controlled real-world evaluation environments, such as regulatory sandboxes or living labs, can support innovation within the boundaries of regulatory frameworks while allowing prospective assessment of performance, safety, and clinical impact in real-world settings^16^. Academic teams will play a central role in designing evaluation protocols within such frameworks to help ensure that deployment is accompanied by rigorous and independent external validation.

Our systematic review shows that a decade of SSU research has largely followed data availability and technical feasibility rather than clinical needs. Deployment, too, has run ahead of a clearly defined clinical purpose and independent evidence of measurable clinical benefit. The operating room is an environment where decisions carry irreversible consequences. Therefore, the question for surgical video AI is not whether it can detect what it has been trained to detect. It is whether what the AI model detects matters to the patient, the surgeon, and to healthcare systems. Answering that question, rigorously and independently, will be at the top of the surgical video AI community’s research agenda.

## Data Availability

No datasets were generated or analyzed during the current study.
